# From Complex Modulus E* to Creep Compliance D(t): Experimental and Modeling Study

**DOI:** 10.3390/ma13081945

**Published:** 2020-04-21

**Authors:** Abdeldjalil Daoudi, Daniel Perraton, Anne Dony, Alan Carter

**Affiliations:** 1 École de Technologie Supérieure (ÉTS), Construction Engineering, 1100 Notre-Dame Ouest, Montreal, QC H3C 1K3, Canada; daniel.perraton@etsmtl.ca (D.P.); alan.carter@etsmtl.ca (A.C.); 2 Institut de Recherche en Constructibilité, Université Paris-Est, 94234 École Spéciale des Travaux Publics, 28 avenue du Président Wilson, 94234 Cachan CEDEX, France; adony@estp-paris.eu

**Keywords:** creep compliance, complex modulus, 2S2P1D model, direct tensile test, direct compression test, indirect tensile test

## Abstract

Creep compliance (D(t)) is a very important input for the thermal cracking resistance in the Mechanistic-Empirical Pavement Design Guide (MEPDG). The aim of the work presented here is to predict the results of creep compliance D(t) from the result of complex modulus E*(ω). The work plan is divided in two main parts: an experimental part consisting of creep tests, and a modeling part. Three configurations were compared together, namely direct tensile, direct compression and indirect tensile tests. The modelling part consists of using a 2S2P1D model coupled to Kopelman approximation to switch from the frequency domain to the time domain. Additionally, 2S2P1D was used to calibrate the generalized Kelvin–Voigt model and get the creep compliance directly from E* results. The experimental results show that D(t) from direct tensile and direct compression are the same in the viscoelastic domain and are greater than D(t) from the indirect tensile test. The indirect tensile test (IDT) seems to be very difficult to achieve compared to the other two variants. The converted results using the 2S2P1D model coupled to Kopelman approximation and the results from the GKV model describe the experimental points very well.

## Highlights

- The use of rheological models is a very simple and good method to predict creep compliance D(t) from complex modulus E(ω).
- Creep compliance from direct tensile and direct compression tests are the same and are more representative to the field.
- The IDT creep test configuration allow to measure parallel creep compliance which is not representative with thermal cracks observed in the real roads.
- Direct creep compliance tests are easier to perform comparing to the IDT configuration proposed by AASHTO T322-07.

## 1. Introduction

The thermomechanical behavior of bituminous asphalt mixes is very complex. According to Benedetto [1] four behaviors are observed depending on the strain amplitude (|ε|) and the number of cycles (N) of the applied loading. For small strain amplitudes (|ε| < 10^−4^) and a few hundred loading cycles, the behavior can be considered as linear viscoelastic (LVE). For tens of thousands of cycles, the phenomenon of fatigue manifests. Rutting is created by the accumulation of irreversible permanent deformations caused by near-breaking amplitudes. The variation of the temperature can cause deformations of a few percent (|ε| >> 10^−4^), the observed behavior is strongly nonlinear.

The viscoelastic linear behavior of asphalt mixes is mainly characterized by two types of tests: quasi-static loading tests in the time domain (relaxation and creep) and cyclic loading tests in the frequency domain (complex modulus). Relaxation is the evolution of the stress in time for the application of a constant deformation, and creep is the evolution of the strain for a constant stress applied. As for the complex modulus, it is the expression of rigidity of viscoelastic materials under cyclic loading; it represents the ratio between the cyclic stress by the cyclic strain.

Laplace–Carson transform is a mathematical tool allowing substituting variables, in particular to go from the time domain to the frequency domain. Because integral equations describing material LVE behavior in a time domain are difficult to apply, the Laplace–Carson transform is used to turn integral equations into algebraic, therefore simplifying calculations (Equation (1)) [1,2].

(1) $$g^{*}\left(p\right)=p\int_{0}^{+\infty }e^{-pt}g\left(t\right)dt$$

where g(t) is a time dependent function, g*(p) is function dependent on p, p is a complex variable, corresponding to time in the transform domain. If p = iω (ω is the angular frequency) we have:

(2) $$E^{*}\left(\omega \right)\times D^{*}\;\left(\omega \right)=1$$

where E* is the complex modulus (relaxation in frequency domain) and D* is the complex compliance (creep compliance in frequency domain).

Creep compliance (ratio between deformations in time by the maximum stress applied) is a very important input for low temperature or thermal cracking module in the Mechanistic-Empirical Pavement Design Guide (MEPDG) software [2,3]. Too low creep may lead to cracking at low temperature and too high creep may lead to high potential of rutting at service temperatures [4]. This type of test (creep) is also a very important element for characterization of bitumen at low temperature; the PG High-Low temperature system is based on a creep test (bending beam rheometer, BBR) for the determination of the low temperature of bitumen [5,6,7,8,9].

The American Association of State Highway and Transportation Officials (AASHTO T322-07) describes a test protocol for the creep compliance [10]. The standard proposes to work in indirect tensile test (IDT) configuration on a cylindrical test specimen of 150 mm in diameter and 38 mm to 50 mm of thickness. The specimen compaction is performed by the gyratory compactor. The test is generally carried out at three different temperatures ranging from 10 °C to −30 °C depending on the grade of the bitumen. A static load is applied along a diametral axis in order to have an indirect tensile stress. The creep compliance is a non-destructive test; the applied loading must always be in the linear viscoelastic domain. However, the load must be high enough to cause sufficient horizontal deformation so the noise in the data acquisition process is insignificant. During the test, vertical and horizontal deformations are measured and the creep compliance represents the time-dependent horizontal deformation by the applied stress, which can be calculated by the given Equation (3):

(3) $$D\left(t\right)=\frac{\Delta X_{tm,t}\times D_{avg}\times b_{avg}}{P_{avg}\times GL}\times C_{cmpl}$$

(4) $$C_{cmpl}=0.6354\times {\left(\frac{X}{Y}\right)}^{-1}-0.332$$

where:
D(t):creep compliance at time t (1/kPa).GL:gauge length in meters (mm).D_avg_:average diameter of all specimens (mm).b_avg_:average thickness of all specimens (mm).Pavg:average creep load (kN).ΔX_tm,t_:trimmed mean of the normalized horizontal deformations of all specimen faces at time t (mm/mm).C_cmpl_:correction factor.$\frac{X}{Y}$:absolute value of the ratio of the normalized, trimmed mean of the horizontal deformations (i.e., ΔX_tm,t_) to the normalized, trimmed mean of the vertical deformations (i.e, ΔY_tm,t_) at a time corresponding to ½ the total creep test time for all specimen faces.


The National Cooperative Highway Research Program (NCHRP) in its report 530 presents a laboratory test program to evaluate and compare measurement of creep compliance using IDT configuration, uniaxial compression, and uniaxial tension tests [11,12]. The objective was to review the test procedure described in AASHTO T322-07. The results show that the behavior is very anisotropic. The compliance measured with the IDT test was lower than the compliance determined in uniaxial compression, which in turn was lower than the uniaxial tension compliance. The NCHRP chose to validate the test procedure described in AASHTO T322-07 because the compliance measured in perpendicular plane in relation to the compaction is generally higher than that determined parallel to compaction. However, we note that thermal cracks at low temperatures are generally perpendicular to the direction of compaction of the pavement. Lowering road temperature creates contraction stress; the contraction is blocked in the longitudinal direction of the road which leads to transverse cracking [4,13]. The IDT configuration allows determining parallel creep compliance contrary to the direct tensile and direct compression configurations. Additionally, we note that compaction procedure with gyratory compactor is not very representative of field compaction.

The viscoelastic theory offers the possibility to get creep compliance results directly from complex modulus results. Olard et al. conducted a study at binder scale for the prediction of BBR results (creep compliance test) from shear complex modulus results (G*) [14]. Complex modulus tests were performed by Métravib (tensile/compression configuration) then the results were converted to shear complex modulus using Poisson’s ratio (|E*| = 2 (1 + ν) |G*|). incompressibility was supposed, so that ν = 0.5 and the approximation |E*| = 3 | G*|was used [4,14]. To change from the frequency domain to the time domain, Olard et al. used four approximate equations namely Kopelman equation (1958), Christensen equation (1982), Ninomiya and Ferry equation (1959) and Schwarzl and Struik equation (1968) [14,15,16]. Creep compliance results obtained from BBR data were compared with the converted results from the approximate equations (prediction). The predicted results describe the experimental data well.

**Figure 1:**
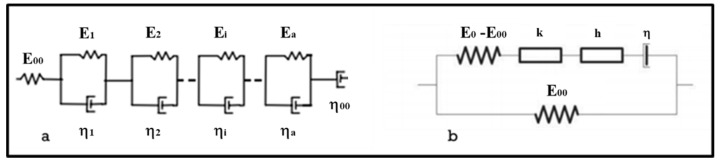
(**a**) The generalized Kelvin–Voigt model (GKV); (**b**) 2S2P1D model.

Any linear viscoelastic body can be modelled by the generalized Kelvin–Voigt model (GKV) (Figure 1a) [17]. This model is an assembly of simple rheological elements like springs and dashpots, the complex modulus and the creep compliance are given by Equations (5) and (6).

(5) $$E^{*}\left(\omega \right)={\left(\frac{1}{E_{00}}+\frac{1}{i\omega \eta_{00}}+\sum_{i=1}^{n}\frac{1}{E_{i}+i\omega \eta_{i}}\right)}^{-1}$$

(6) $$D\left(t\right)=\frac{1}{E_{00}}+\sum_{i=1}^{n-1}\frac{1}{E_{i}}\;\left(1-e^{\frac{t}{\tau_{i}}}\right)+\frac{1}{\eta_{00}}$$

where:
E*(ω):complex modulus (MPa).E_00_ and η_00_value of the complex modulus and the viscosity when ωτ → 0.ω:is the angular frequency, so that ω/2π is equal to the frequency.η_i_ and E_i_:viscosity and elastic modulus of each Kelvin Voigt branch.D(t):creep compliance (1/MPa).τ:is the relaxation time for single branch.


The unidimensional rheological model with continuous spectrum called 2S2P1D (Figure 1b) [18] is widely used for the prediction of the LVE behavior of bituminous materials; it consists of two elastic (spring) elements, two parabolic creep elements and one viscous (dashpot) element. The complex modulus is given by the Equation (7). However, no analytical expression of the creep compliance function of this model is available in the time domain.

(7) $$E^{*}\left(\omega \right)=E_{00}+\frac{E_{0}-E_{00}}{1+\delta {\left(i\omega t\right)}^{-k}+{\left(i\omega t\right)}^{-h}+{\left(i\omega \beta t\right)}^{-1}}$$

where:

E_0_ is the value of the complex modulus when ωτ →∞.
δ, k and h are dimensionless constants and β is a dimensionless parameter, related to Newtonian viscosity η.

Several studies present a method to get viscosities (η_i_) and modulus (E_i_) of the generalized Kelvin–Voigt (GKV) model from 2S2P1D model [19,20,21]. After the calibration of the 2S2P1D model in the frequency domain (E*), the method consists of minimizing the difference between the GKV model results and the results given by a reference viscoelastic model (2S2P1D).

## 2. Methods and Materials

### 2.1. Methods

The objective of this study is to predict the results of creep compliance in time domain D(t) directly from the results of complex modulus.

**Figure 2:**
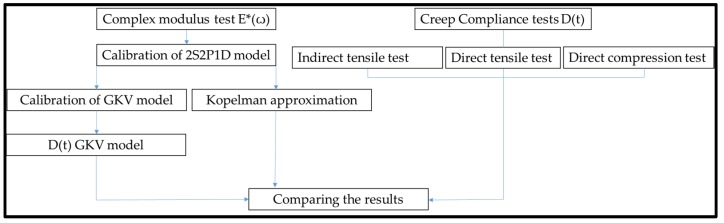
The work plan.

The methodology is divided into two main parts: an experimental part and a modelling part. The experimental part consists of realization of complex modulus tests and creep compliance tests (Figure 2).

Complex modulus tests were performed in traction-compression configuration according to Quebec’s standard LC26-700. Seven test temperatures ranging from −20 °C to 40 °C with a step of 10 °C and a frequency sweep from 0.1 Hz to 10 Hz were used. The imposed strain was ±50 µstrain. Complex modulus results were fitted using 2S2P1D model, then two different methods were used to switch from the frequency domain to the time domain for creep compliance. First, 2S2P1D results were coupled with the approximation proposed by Kopelman which is mathematically simpler than the other three equations proposed in Olard’s et al. [14] study. Second, the GKV model was calibrated according to the minimization with 2S2P1D model proposed by Tiouajni et al. [19]. and the creep compliance in time domain is given by Equation (6).

Creep tests were performed using three configurations namely: direct tensile configuration, direct compression configuration and indirect tensile configuration. Direct tensile and direct compression tests were achieved on cylindrical specimens with a diameter of 75 ± 1 mm and a height of 135 ± 2 mm. A static traction or compression stress load was applied on the samples for 300 s; it took 0.1 s to reach the imposed load, without impact, from a contact force of 50 N. The measurement of deformation was insured by three extensometers of 25 mm in length. Four test temperatures were used ranging from −20 °C to 10 °C with a step of 10 °C. The amplitude of the imposed stress was fixed by taking in to account complex modulus results. For each test temperature, before to start the creep test and at the end of it, a partial complex modulus test was done in order to check if the material was still in the viscoelastic behavior domain. Indirect tensile creep tests were performed on cylindrical samples with a diameter of 150 mm and a thickness of 38 mm. AASHTO T322-07 has not set a load application speed or a maximum amplitude, so, in order to have the same test parameters, the IDT loading was applied in 0.1 s and the amplitude was fixed manually in order to have a minimum 50 µstrain at 0.1 s.

### 2.2. Materials

**Figure 3:**
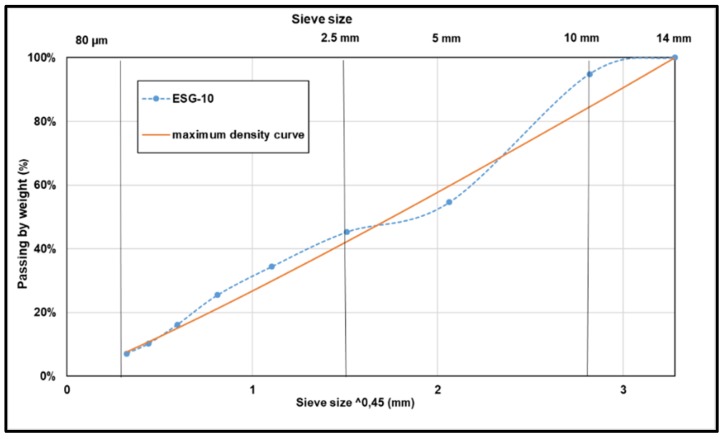
The gradation of asphalt mix (ESG-10).

A 0–10 mm surface mix, namely ESG-10, was used. The gradation is given in Figure 3. The bitumen is an unmodified PG 58-34, the penetration and the ring and ball temperature of this bitumen are respectively 153 ± 1, 1/10 mm and 43 ± 0.1 °C. The mixing temperature used was 150 °C, and the compaction temperature 135 °C. The aggregates were heated 15 °C more than the bitumen as indicated in the LC (26-003) test method, i.e., 165 °C.

**Figure 4:**
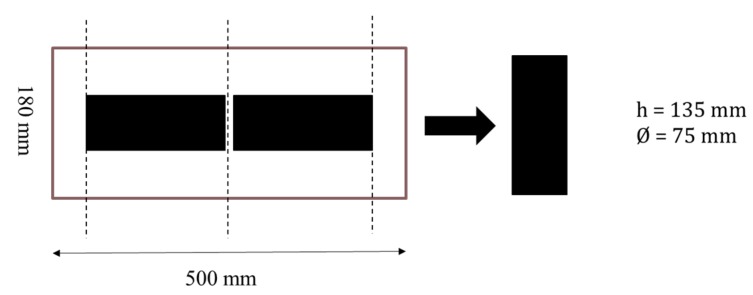
Sample preparation for complex modulus test, direct tensile test and direct compression test.

**Figure 5:**
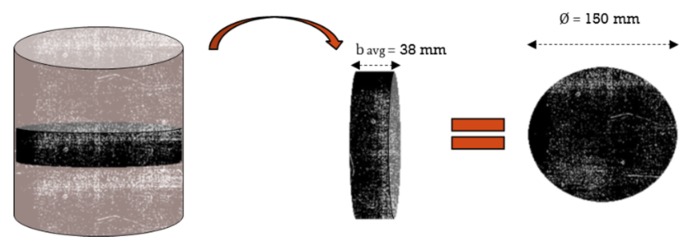
Sample preparation for indirect tensile test.

The cylindrical samples for complex modulus tests, direct tensile and direct compression creep tests were cored from slabs (100 mm × 180 mm × 500 mm) compacted in laboratory using MLPC (*Materiel Laboratoire Ponts et Chaussées*) wheel compactor according to Quebec’s LC test method (Figure 4). For indirect tensile creep tests, the specimens were cut from cylindrical samples compacted by gyratory compactor (Figure 5). Before doing tests, the air voids content was measured using hydrostatic weighing and the maximum density of asphalt mix (D_mm_).

## 3. Results and Discussion

### 3.1. Complex Modulus Results and 2S2P1D Calibration

**Figure 6:**
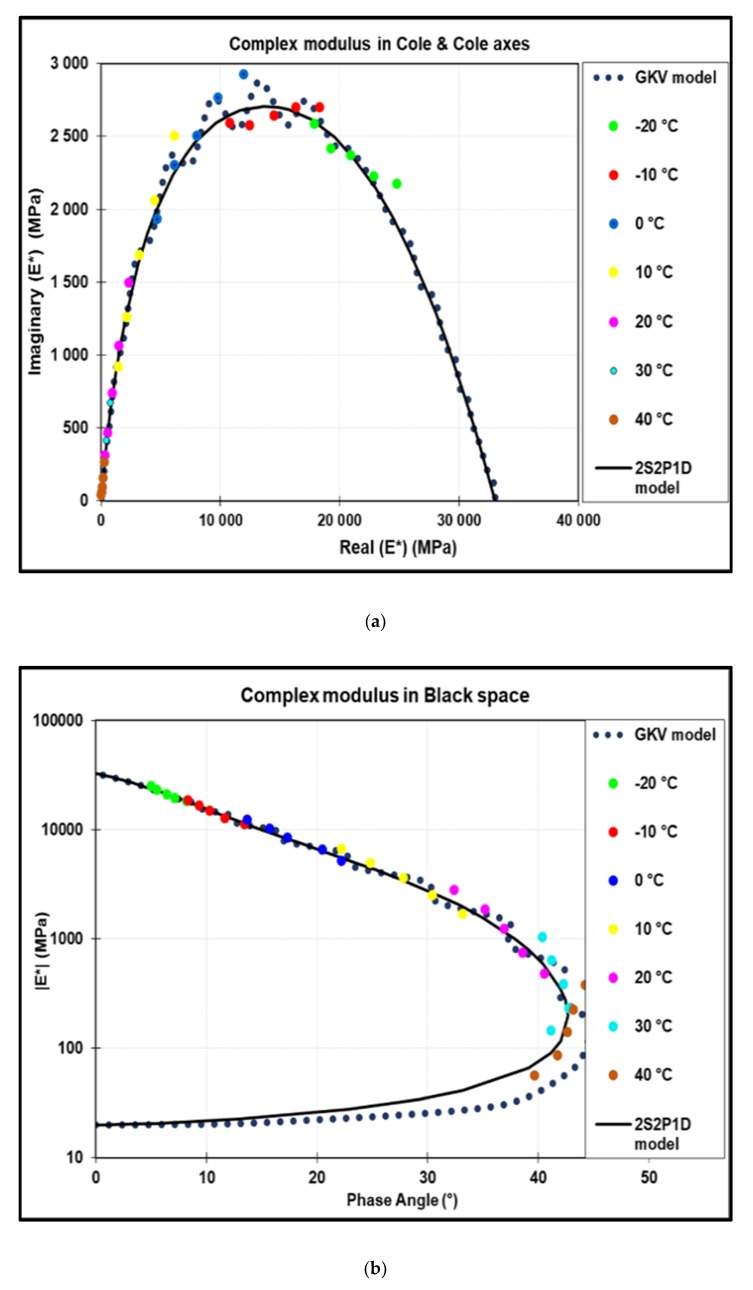
Complex modulus results in (**a**) Cole–Cole representation and (**b**) black space.

**Figure 7:**
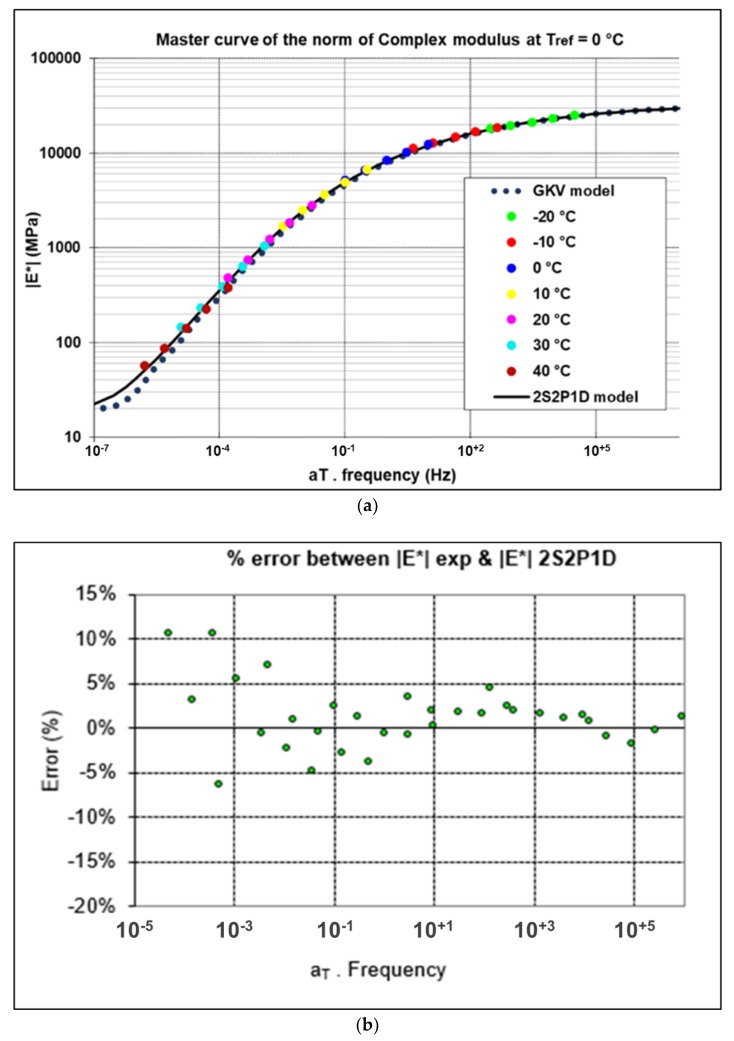
(**a**) Master curve of complex modulus. (**b**) the error between 2S2P1D model and the experimental points.

Complex modulus results are presented in Figure 6 and Figure 7. The calibration of the 2S2S1D model was performed in Cole–Cole curve and black space in order to fix the shape parameters (k, h, δ and β). Asymptotic moduli E_00_ and E_0_ were fixed by a linear extrapolation respectively at very low frequencies and very high frequencies. Characteristic time τ depends on temperature as follows:

(8) $$a_{T}=\frac{\tau }{\tau_{0}}$$

where τ_0_ is the characteristic time at the reference temperature (T_ref_) and a_T_ is the shift factor. a_T_ values may be approximated by the Williams–Landel–Ferry (WLF) equation (Equation (9)) when Time-Temperature superposition is valid [22,23,24].

(9) $$\log(a_{T})=\frac{-C1\;\left(T-Tref\right)}{C2+\left(T-Tref\right)}$$

where C1 (23.90) and C2 (148.90) are empirical constants depending on the material. The calibration of the GKV model was performed according to the minimization proposed by [19,20], 19 branches of Kelvin–Voigt model were used.

Master curve is presented in Figure 7 at T_ref_ = 0 °C. As it is noted, the 2S2P1D model and GKV model describe very well the experimental points. The error between the experimental points and 2S2P1D model are presented in Figure 7. The majority of the points are located within an error interval of ± 5% for the norm of complex modulus (|E*|) and ± 2° for phase angle (φ). The maximum error noted is 10.5% for |E*| and 3.3° for φ. The maximum error between the GKV model and the 2S2P1D model is 14% for |E*| and 3° for φ.

The calculation of creep compliance is done using two different methods. First, using the GKV model, the creep compliance of this model is given by Equation (6). The second method consists of coupling the 2S2P1D results with the approximation proposed by Kopelman. The 2S2P1D model doesn’t have a creep function in frequency domain while using Kopelman Equation (15) can allow to switch from the frequency domain to the time domain. The explanations of the calculations are given below:

(10) $$E^{*}=E_{1}+iE_{2}$$

(11) $$D^{*}=D_{1}-iD_{2}$$

(12) $$E^{*}\left(\omega \right)\times D^{*}\left(\omega \right)=1$$

(13) $$D_{1}=\frac{E_{1}}{E_{1}^{2}+E_{2}^{2}}$$

(14) $$D_{2}=\frac{E_{2}}{E_{1}^{2}+E_{2}^{2}}$$

(15) $$D\left(t\right)={\left|D\left(\omega \right)\right|}_{\omega =1/t}$$

where:

E_1_: the real part of the complex modulus (storage modulus) (MPa).
E_2_: the imaginary part of the complex modulus (loss modulus) (MPa).
D_1_: the real part of the complex compliance (1/MPa).
D_2_: the imaginary part of complex compliance (1/MPa).
i: the imaginary unit.

**Figure 8:**
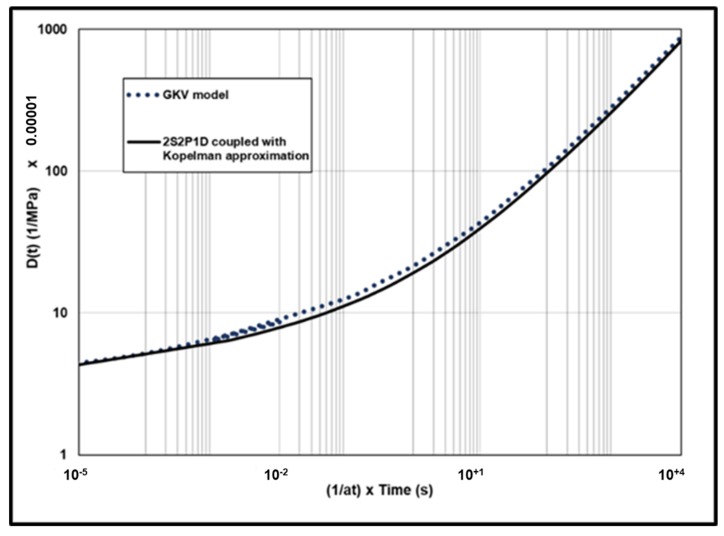
Creep compliance predicted with the GKV model and with the 2S2P1D coupled to Kopelman approximation.

Creep compliance from the GKV model and from the 2S2P1D model coupled with Kopelman approximation are presented in Figure 8. The two curves are very close. The error is calculated from 10^−5^ s to 10^+5^ s using Equation (16).

(16) $$\%\;Error=\frac{D{\left(t\right)}_{2S2P1D+Kopelman}-D{\left(t\right)}_{GKV}}{D{\left(t\right)}_{2S2P1D+Kopelman}}$$

**Figure 9:**
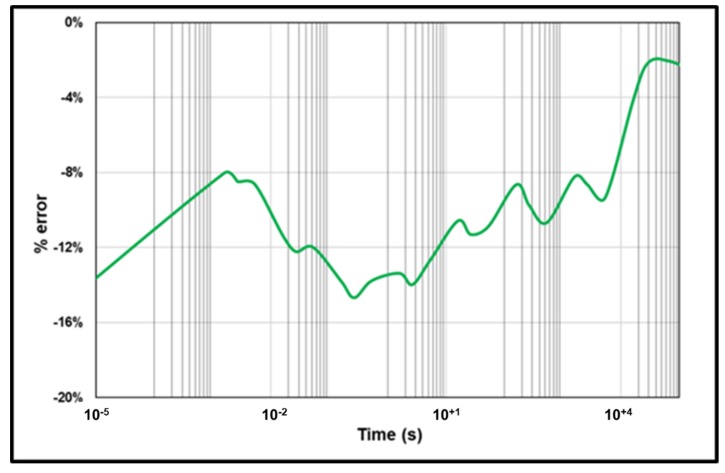
The error between D(t)GKV model and D(t) from 2S2P1D coupled to Kopelman approximation.

The results are presented in Figure 9. The error is between −2% to −15%.

### 3.2. Direct Tensile and Direct Compression Creep Tests Results

**Figure 10:**
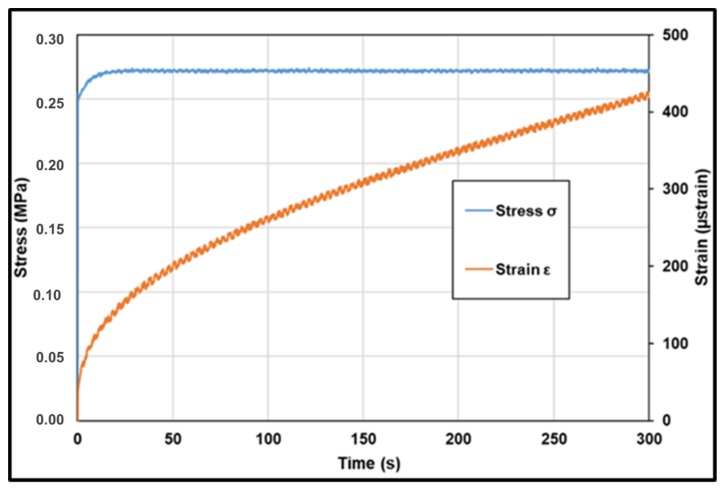
Creep loading for direct tensile and direct compression tests.

Creep tests were performed using three configurations namely direct tensile test, direct compression test and indirect tensile test. As shown in Figure 10, for direct tensile tests and direct compression tests, a static stress was applied to the cylindrical samples. This configuration allows to have a homogeneous stress distribution, so the creep compliance can be calculated directly using the Equation (17).

The load was applied in 0.1 s. The amplitude of force and stress applied are presented in Table 1. For the first 10 to 15 s, the applied load was not stable. Creep compliance was calculated (Equation (17)) when the load is stabilized at ± 2% of the target value at t equal to 0.1, 1, 3, 10, 30, 100 and 300 s.

(17) $$D\left(t\right)=\frac{\varepsilon \left(t\right)}{\sigma_{max}}$$

**Figure 11:**
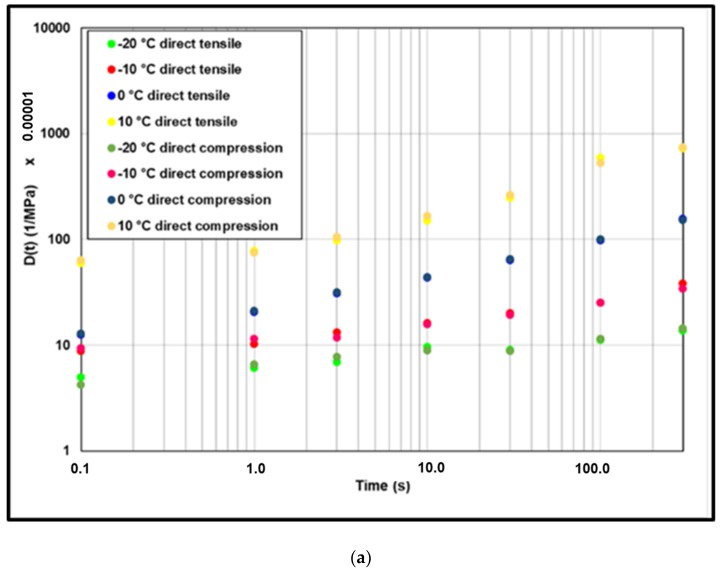

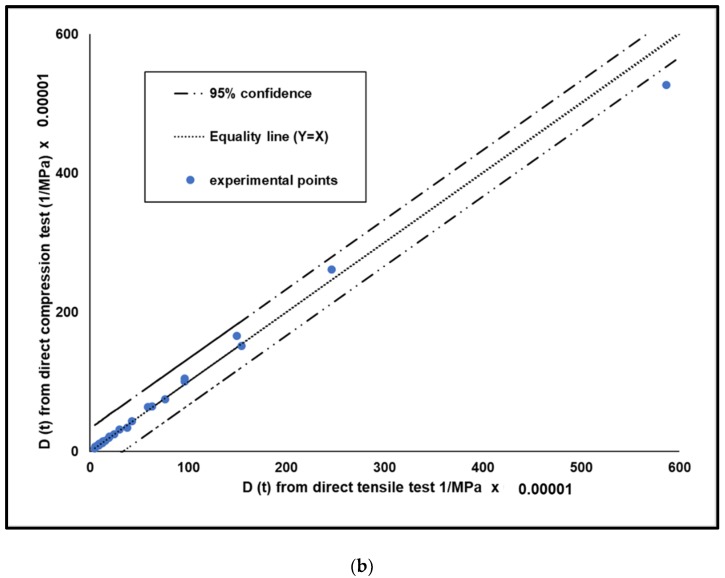
(**a**) Results of direct tensile and direct compression creep tests and (**b**) error analysis.

The results of creep compliance are presented in Figure 11 at different test temperatures (isotherms at −20 °C, −10 °C, 0 °C and 10 °C). According to the results, we notice that the creep compliances from direct tensile and from direct compression are the same. The majority of points are superposed on the equality line with an error interval between ±3% and a maximum error of 18% at high creep compliance value.

This results are contradictory with the results presented by NCHRP [10]. The explanation could be that the results presented by NCHRP were out of the VEL domain. As mentioned before, to verify that our results are inside the linear viscoelastic domain, partial complex modulus tests were performed before and after direct traction and compression tests. Exceeding the viscoelastic limit is characterized by a loss of |E*|. Table 2 presents the percentage of loss of |E*| at test temperatures and frequencies. These percentages were calculated using Equation (18). The non-significant losses observed confirm that viscoelastic limit was not exceeded.

(18) $$\%\;of\;loss=\frac{\left|E_{before\;direct\;creep\;tests}^{*}\right|-\left|E_{after\;direct\;creep\;tests}^{*}\right|}{\left|E_{before\;direct\;creep\;test}^{*}\right|}$$

**Figure 12:**
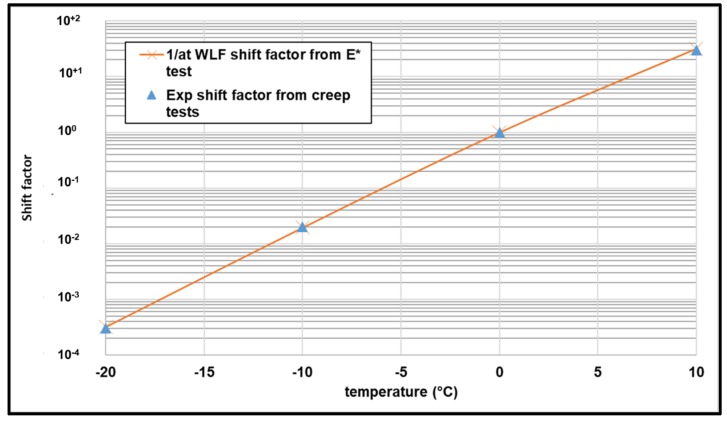
EXP shift factor vs. the inverse of Williams–Landel–Ferry (WLF) equation.

The application of the time temperature superposition principle (TTSP) allows having a unique curve. The WLF equation is widely used to determine the shift factor (a_t_) for bituminous materials. However, this shift factor is used in the frequency domain. In order to apply the TTSP to creep compliance results, two methods are used to determine the shift factor in time domain. First a graphical method is used (experimental: EXP) and second the inverse of a_t_ WLF calculated from complex modulus test were used. The results are presented in Figure 12. It can be noted that the graphical exp shift factor can be approximated by the inverse of WLF equation.

**Figure 13:**
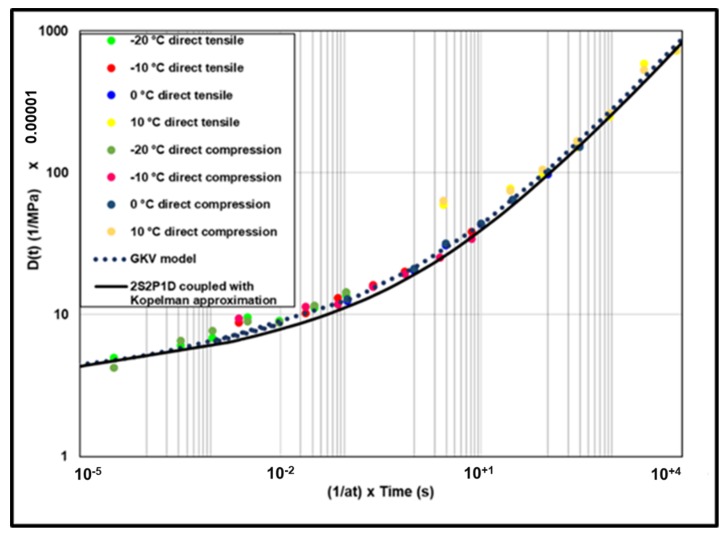
Master curve of creep compliance at 0 °C.

The master curve built with the experimental points is presented at 0 °C (Figure 13) comparatively with the creep compliance predicted with GKV model and 2S2P1D model coupled to Kopelman approximation. Both of models seem to describe very well the experimental points. These results confirm that it is possible to predict creep compliance (static load test) results directly from complex modulus (cyclic load test) results.

### 3.3. Indirect Tensile Creep Test Results

**Figure 14:**
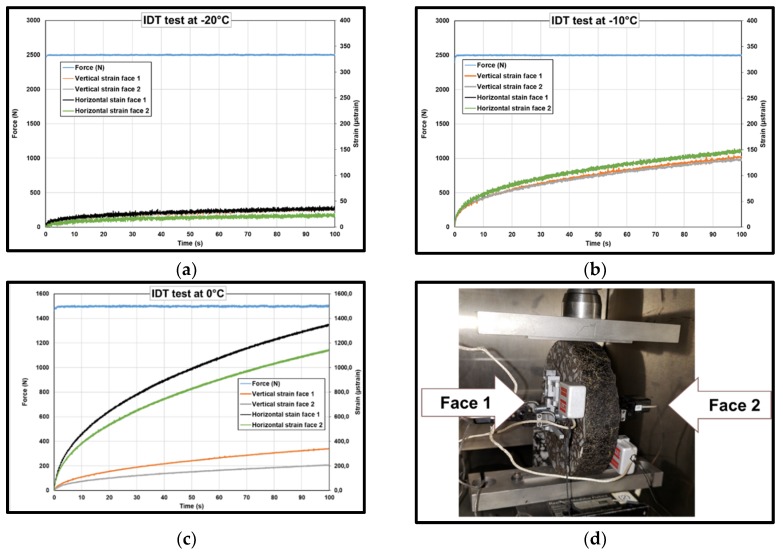
Creep loading for indirect tensile test at (**a**) −20 °C, (**b**) −10 °C, (**c**) 0 °C, (**d**) IDT configuration

IDT creep tests were carried out by applying a static load on a diametral axis on cylindrical specimens. The deformations were measured on both sides of the specimens. The applied loads as well as the deformations on both faces are presented in Figure 14.

The difference in strain measured on both sides increased by increasing test temperature. At −20 °C the variation of horizontal strain between face 1 and face 2 was 2% ± 5% and for vertical strain the difference was 30% ± 10%. At −10 °C the gap for horizontal strain was 4% ± 3% and vertical strain it was 5% ± 1%. Finally, for 0 °C the difference was very significant for horizontal strain 37% ± 3% and 16% ± 1% for vertical strain. Additionally, it can be noted that the maximal vertical strain at 0 °C was greater than the limit of viscoelastic domain found in the literature. However, because of the shape of the specimen, it was not possible to test the complex modulus before and after the static load. The gap between strains measured on both faces can be an indicator that the viscoelastic limit was exceeded; for that reason, the test was stopped after 100 s and not carried out at 10 °C.

**Figure 15:**
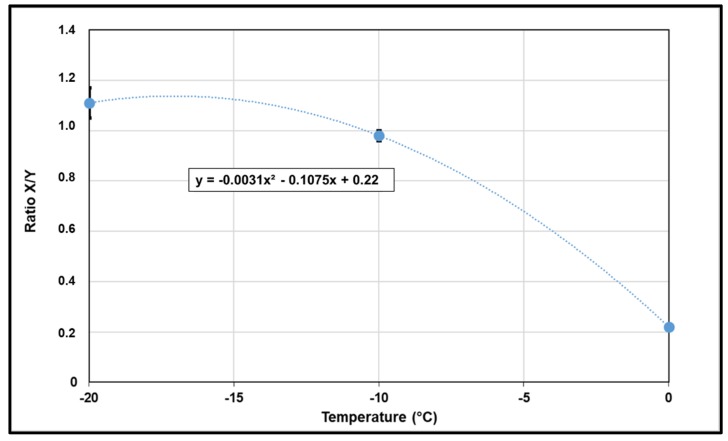
X/Y vs. test temperature.

The ratio between the mean horizontal strain (X) and the mean vertical strain (Y) was measured, the results are presented in Figure 15. The points follow a polynomial law of order two in the range of temperature tested.

**Figure 16:**
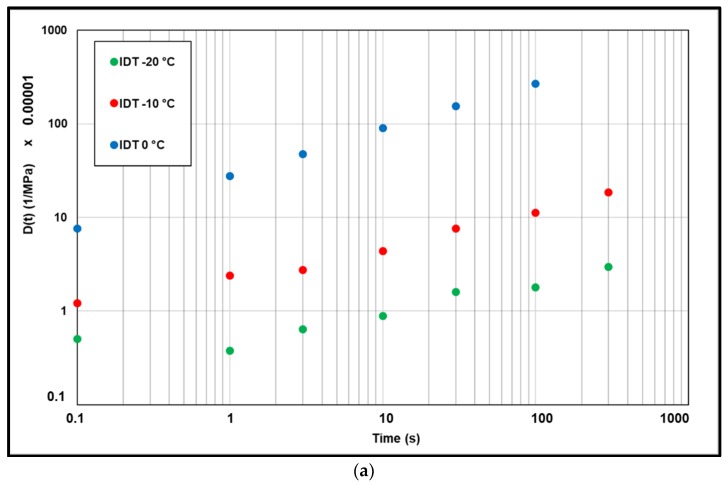

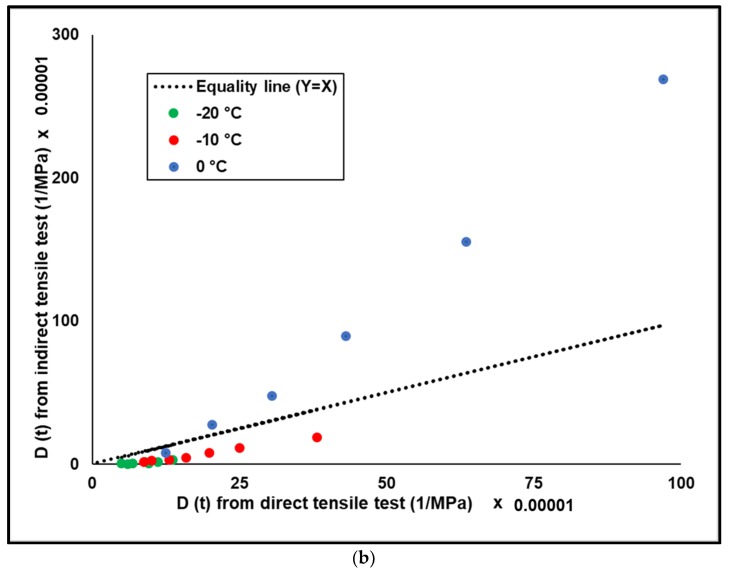
(**a**) Isotherms of IDT creep compliance. (**b**) IDT creep compliance vs. direct creep compliance.

IDT creep results were calculated using the equation proposed by AASHTO. The results (isotherms) are presented in Figure 16. The values appear lower than direct tensile (DT) and direct compression (DC) tests except for the test points at 0 °C, which may confirm that the linear viscoelastic limit was exceeded. At −20 °C, creep compliance from IDT test was lower than direct tensile and direct compression creeps with factor ranging between 15.0 to 1.05. At −10 °C there is a factor of 3.0 to 1.05 with two points at 0.85. At 0 °C, the factor ranges from 0.75 to 0.35.

**Figure 17:**
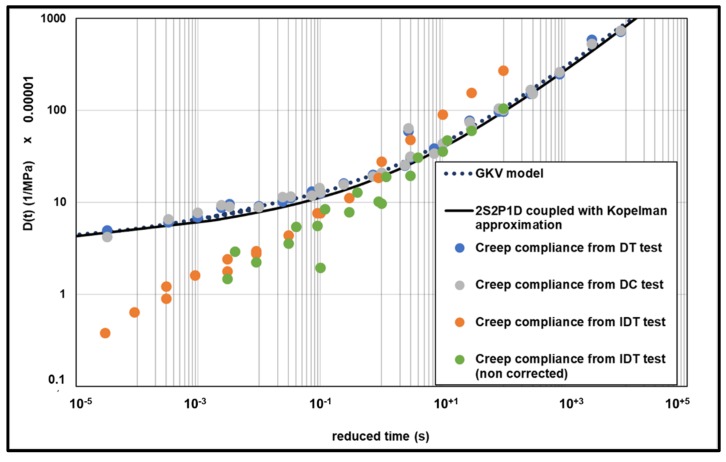
Master curve of creep compliance at 0 °C.

The master curves of IDT creep compliance test are presented at 0 °C in Figure 17 where the creep compliance from GKV model and the creep compliance from the 2S2P1D model coupled to Kopelman approximation and from direct tensile and compression creep test are compared. To build the master curve for IDT, test shift factors were determined graphically and seem to be ten times bigger the inverse of WLF law using the same C1 and C2 from complex modulus test. It is difficult to know the reason of that, C1 and C2 are empirical constants depending on the material. However, this may be related to the correction factor proposed by the AASHTO.

The results show that creep compliance determined from IDT (corrected) test appears lower than the creep predicted from GKV model and 2S2P1D model coupled to Kopelman approximation except for the points determined at 0 °C. This could be explained by the fact that the viscoelastic domain was exceeded and therefore the creep was much more pronounced in this case. Additionally, Figure 17 shows the non-corrected IDT creep compliance (without applying C_cmpl_). In this case, to build the master curve, the shift factors were determined using the inverse of WLF law.

## 4. Conclusions

The objective of the work presented here was to predict creep compliance D(t) results from complex modulus E*(ω) results. Several methods are proposed in the literature allowing to go from the frequency domain to the time domain and vice-versa such as the inverse of the transform of Carson-Laplace and by more or less complex numerical methods. Rheological models, if properly calibrated, seem to be a very good and simple method to go from E* (cyclic test) to D(t) (static test). To achieve this objective, the proposed working method consists of two main parts: an experimental part and a modelling part. For the experimental part, two types of test were performed namely: cyclic test–E*(ω) and creep static test–D(t). Complex modulus tests were done using direct tensile and compression tests. For creep test, a comparison was done between indirect tensile configuration (proposed by AASHTO) and direct tensile and direct compression tests.

The results show that D(t) from direct tensile and direct compression tests are the same in the viscoelastic behavior domain and are greater than D(t) from indirect tensile test. This can be explained by the direction of the measured creep modulus. D(t) perpendicular to the direction of compaction are higher compared to the parallel one. Compared to the field, the cracks observed by lowering of temperature are in an axis perpendicular to the direction of compaction of the roads. Moreover, the compaction method proposed by AASHTO (with the gyratory compactor) is not very representative compared to compaction on site. For direct tensile and direct compression tests, the samples were compacted using MLPC wheel compactor. Additionally, it is complicated to compare creep test in IDT configuration to direct compression or tensile tests. For IDT, the results show significant difference between the strain measured in both faces. Further, it’s very difficult to know if the load applied remains inside the viscoelastic behavior domain for IDT configuration. The proposed method for converting E*(ω) to D(t) consists in using existing rheological models. 2S2P1D is very commonly used for modelling of asphalt materials but this model does not have a creep function in time domain. To change from the frequency domain to the time domain, we propose to couple the results from 2S2P1D to the approximation proposed by Kopelman. The generalized Kelvin–Voigt model can predict the creep compliance in time domain, but it is more difficult to calibrate. A minimization method is proposed in literature to calibrate GKV model from 2S2P1D model and therefore having D(t) results directly from E* results. The two methods were compared, and the results show a very good concordance between them.

## Figures and Tables

**Table 1:** Forces and stresses amplitude applied for direct tensile and direct compression tests.

Temperature (°C)	Force Max (N)	Stress Max (MPA)
−20	1800	0.41
−10	1500	0.34
0	1200	0.27
10	500	0.11

**Table 2:** calculation of loss in |E*|.

	Modulus Loss			
	**−20 °C**	**−10 °C**	**0 °C**	**10 °C**
**10 Hz**	0.32%	0.02%	−0.13%	1.50%
**3 Hz**	0.19%	0.51%	0.51%	2.41%
**1 Hz**	0.39%	0.53%	0.17%	2.11%
**0.3 Hz**	0.05%	0.00%	−1.37%	2.17%
